# The interplay between inflammation, physical activity and metabolic syndrome in a remote male geriatric community in Southern Taiwan: the Tianliao Old People (TOP) study 03

**DOI:** 10.1186/1758-5996-5-60

**Published:** 2013-10-14

**Authors:** Chia-Ling Chang, Po-Tseng Lee, Wei-Ting Chang, Chin-Sung Chang, Jyh-Hong Chen, Liang-Miin Tsai, Chih-Hsing Wu, Ping-Yen Liu

**Affiliations:** 1 College of Medicine, National Cheng Kung University, Tainan, Taiwan; 2 Division of Cardiology, Internal Medicine, National Cheng Kung University Hospital, Tainan, Taiwan; 3 Institute of Clinical Medicine, National Cheng Kung University, Tainan, Taiwan; 4 Internal Medicine, National Cheng Kung University Hospital Dou-Liou Branch, Yun-Lin County, Taiwan; 5 Department of Family Medicine, National Cheng Kung University Hospital, Tainan, Taiwan

**Keywords:** Metabolic syndrome, Inflammation, Physical activity, Geriatric

## Abstract

**Background:**

Both physical activity and inflammation are important in the pathophysiology of metabolic syndrome (MetS). Our study aim is to explore their associations in an elderly male (≥ 65 years old) cohort.

**Methods:**

We enrolled 309 elderly male residents (mean age: 74.4 ± 6.0 years) in a remote southern Taiwan community. The physical activity was recorded by a standard questionnaire. A high-sensitivity C-reactive protein (hsCRP) level > 3.0 mg/L indicated a high inflammatory status.

**Results:**

The total prevalence rate of MetS was 27.8% in this male geriatric cohort. Median hsCRP levels were significantly higher in the MetS group (1.60 ± 0.7 vs. 1.0 ± 0.3 mg/L, *p* < 0.01), and the risk of elevated hsCRP increased with escalating MetS components (*p* for trend < 0.001). The non-MetS group had higher amount of median weekly physical activity (183.1 ± 19.0 vs. 173.5 ± 10.6 MET-hr/week, *p* = 0.029), which was also higher among those with lower hsCRP levels (186.1 ± 14.1 vs. 167.8 ± 38.5 MET-hr/week, *p* = 0.013). Multivariate analysis showed that higher body mass index (ORs: 1.527, 95% CI: 1.319-1.768, *p* < 0.01) insulin (OR: 1.128, 95% CI: 1.045-1.218, *p* < 0.01) and physical activity (ORs: 0.997, 95% IC: 0.994-0.999, *p* < 0.05) were independent predictors of MetS, but not hsCRP level (*p* > 0.05).

**Conclusions:**

Reduced physical activity was one major pathophysiological MetS factor in our Asian geriatric participants.

## Introduction

In 1988, the concept of metabolic syndrome (MetS), a cluster of metabolic factors, was proposed [1]. MetS is not a single disease, but a syndrome that causes physiological abnormalities such as abnormal vascular function, impaired glucose tolerance, and abnormal coagulation factors, all of which increase the risk of poor outcomes for diabetes mellitus (DM) and cardiovascular disorders [2,3]. Obesity [4,5], physical activity [6-8], and inflammation [9,10] may be the major pathophysiological factors of MetS. It is still unclear that the association is true in the elderly (≥ 65 years old). We thus explored the association between MetS, physical activity, and low-grade inflammation markers, presented by high-sensitivity C-reactive protein (hsCRP) concentrations in elderly men in a remote area of southern Taiwan.

hsCRP is one indicator of inflammation. When developing inflammation, plasma hsCRP level elevates abruptly; therefore, it is referred to as a biomarker of low-grade inflammation. In addition, adipose tissue releases many inflammatory cytokines, such as interleukin-6 and tumor necrosis factor-α, which elevate plasma hsCRP levels [9]. Aging process also elevates plasma hsCRP levels [9,11]. Higher plasma hsCRP levels are associated with an increased the risk of DM [10,12-15] and cardiovascular disease [10,11,13,16-18]. Plasma hsCRP levels are also related to MetS [9,10,13,15,19,20] and the components of MetS [19].

Lower physical activity in healthy young adults is associated with abnormal metabolic consequences such as decreased insulin sensitivity and increased abdominal fat [21], which links many components of MetS. Inflammation also induces insulin resistance [21]. A higher-level physical activity was reported to be associated with decrease plasma hsCRP level [22], which is an indicator of lower inflammatory status. To our best knowledge, it remained unclear whether the association between hsCRP and the strength of daily physical activity regarding MetS is true in the elderly population. We thus hypothesized that though the elderly population might have average higher levels of lower-grade inflammation status indicated by hsCRP levels, higher physical activity could be related to less abnormal metabolic profiles in an aging community survey.

## Methods

### Study population and data collection

According to the public health database in 2010 from the National Health Research Institute in Taiwan, Tianliao was the third-fastest aging village in Taiwan and the highest percentage of elderly residents in southern Taiwan. In addition, the village had only limited medical resources. According to the August 2010 census [23], there were 8,421 people (4,583 men; 3,658 women) in the village and the rate of people older than 65 years of age was 10.5% in Taiwan, but 24.1% in Tianliao. In addition, based on the aging index, defined by equation as the ratio of ([(population ≥ 65 years old)/(population ≤ 14 years old)] × 100), was as high as 299%, Tianliao could be classified as a “severely aging village”.

The study surveyed voluntary men residents over ≥ 65 years old in this remote aging community in July and August 2010. According to the census, there were 1,012 men ≥ 65 years old in Tianliao. Those who wished to participate in the study were called to the nearest public health examination station via the public address broadcasting system or door-to-door invitation. Except for 62 men who were disabled and 269 men who did not live there, only 681 of those recruited were eligible, 414 men agreed to participate in our study. The response rate was 60.8% and the statistical power was 0.80. There were no significant differences in background or age distribution between participants and non-participants. Each participant underwent a structured questionnaire interview and a blood test. The study protocol was approved by the National Cheng Kung University ethics committee (IRB no: ER-99-111), and all participants signed an informed consent form before the study began.

### Questionnaire design

A standardized face-to-face interview based on structured questionnaires was conducted by well-trained interviewers. The questionnaire asked about participant’s medical history, as well as physical activity.

### Physical activity

Self-reported physical activity in usual seven days was calculated through the Taiwanese version of short form of international physical activity questionnaire (IPAQ). The total hours over the last seven days spent on vigorous and moderate-intensity physical activity, walking and sitting was obtained through IPAQ. The amount of physical activity was converted to metabolic equivalent task hours per week (MET-hr/week) according to the IPQA scoring protocol [24]. The formula we applied for the average MET score were calculated from each type of physical activity: vigorous physical activity = 8.0 METs, moderate physical activity = 4.0 METs, walking = 3.3 METs, sitting = 1 MET, and total physical activity = sum of vigorous + moderate + walking + sitting MET-hr/week scores.

### Definition of MetS and DM

MetS was defined, based upon the National Cholesterol Education Program Adult Treatment Panel III (NCEP-ATPIII) with criteria modified in 2005 for Asian Americans [25], as presenting 3 or more of the following components: (1) waist circumference ≥ 90 cm for men; (2) triglycerides ≥ 150 mg/dL; (3) HDL-C < 40 mg/dL for men; (4) blood pressure ≥ 130/85 mmHg or current use of antihypertensive medication; (5) fasting glucose ≥ 100 mg/dL. DM was defined according to the American Diabetes Association 2010 diagnostic criteria [26] and the medical history reported by each participant.

### Laboratory methods

After the participants had fasted for at least 8 hours, peripheral blood samples were collected from them and centrifuged at 3,000 rpm for 15 min at 4°C. The samples were frozen and then sent to a central laboratory for analysis. Measurement of insulin, fasting glucose, hemoglobin A1c (HbA1_C_), total cholesterol, high-density lipoprotein (HDL-C), low-density lipoprotein (LDL-C), and triglyceride levels were described by previous study [8]. Level of hsCRP was measured using a particle-enhanced turbidimetric assay. In this study, we used the cut-off value of hsCRP to predict high level of inflammation by the criteria set by the American Heart Association [27], which defines a plasma hsCRP level > 3.0 mg/L to be as a high hsCRP level.

### Statistical analysis

Data management and statistical analyses were performed with SPSS 12.0. Continuous variables with a normal distribution (fasting glucose, total cholesterol, HDL-C, LDL-C, waist circumference, height, weight, body fat, and body mass index (BMI)) were expressed as means ± standard deviation (SD). Continuous variables with a non-normal distribution (age, hsCRP level, insulin, triglycerides, components of MetS, physical activity, and quantity of alcohol consumed) were expressed as means ± standard error (SE). Categorical variables were reported as numbers and percentages. Normality was tested using the Kolmogorov-Smirnov test. Continuous variables were compared using a Student’s *t* test for normally distributed values; otherwise, a non-parametric Mann–Whitney U test was used. Comparisons between categorical variables were made using a χ^2^ test. Spearman partial correlation coefficients of hsCRP and components of MetS were calculated. Odds ratios (ORs) and 95% confidence intervals (CI) were assessed using logistic regression. Statistical tests were 2-sided; significance was set at *p* < 0.05.

## Results

We enrolled 309 participants (all gentlemen; mean age: 74.4 ± 6.0 years; range: 65–98 years) for analysis. Excluded were 9 with various treatment strategies and 96 with a history of DM. The prevalence of high blood pressure was 76.7%; the mean waist circumference was 85.6 ± 11.0 cm, HDL-C was 47.8 ± 11.6 mg/dL, and fasting glucose was 95.9 ± 9.3 mg/dL; and the median hsCRP was 1.3 ± 0.3 mg/L, weekly physical activity was 179.53 ± 14.14 MET-hr/week and triglyceride level was 97.0 ± 3.5 mg/dL. Table 1 showed the clinical characteristics stratified by MetS among the elderly male population.

**Table 1:** Clinical characteristics: comparison between non-MetS and MetS groups

**Variation**	**Non-MetS (n = 223)**	**MetS (n = 86)**
Age (years)*	74 ± 0.4	74 ± 0.6
hsCRP (mg/L)*	1.0 ± 0.3	1.6 ± 0.7**
ATP III MetS criteria (%)		
Hypertension	71.7	89.5**
Increased waist	18.8	74.4***
Low HDL-C	9.9	70.9***
Hyperglycemia	21.5	59.3***
High triglyceride	3.6	51.2***
Fasting glucose (mg/dL)	93.7 ± 7.7	101.5 ± 10.6***
Insulin (uIU/mL)*	4.8 ± 0.3	9.4 ± 1.4***
Cholesterol (mg/dL)	194.5 ± 36.2	194.1 ± 33.7
Triglycerides (mg/dL)*	84.0 ± 2.1	151 ± 9.0***
LDL-C (mg/dL)	124.8 ± 31.0	123.0 ± 31.7
HDL-C (mg/dL)	51.4 ± 11.1	38.5 ± 6.7***
Waist (cm)	82.2 ± 10.3	94.2 ± 7.6***
Height (cm)	160.9 ± 5.9	161.8 ± 5.4
Weight (kg)	59.5 ± 8.2	69.4 ± 8.3***
BMI (kg/m^2^)	23.0 ± 2.7	26.5 ± 2.8***
Body fat*	18.8 ± 6.2	23.3 ± 0.6***
Physical activity(MET-hr/week)	183.1 ± 19.0	173.5 ± 10.6*
MetSC*	1 ± 0.05	3 ± 0.07***
Smoke, current	17	17.4
Liver disease (%)	3.6	4.7
Stroke (%)	3.1	10.5*
Heart disease (%)	13.0	18.6
Arrhythmia (%)	2.2	3.5
Cardiac catheterization (%)	4.0	4.7

1. Continuous variables with normal distribution are mean ± standard deviation (SD) and with non-normal distribution (variation*) are median ± standard error (SE). Categorical variables are reported as numbers and percentages, as indicated.

2. Continuous variables with normal distribution were tested using Student’s *t* test; otherwise, Mann–Whitney U test was used. Comparisons between categorical variables were made using a χ^2^ test. *p <0.05; **p <0.01; ***p <0.001.

*Abbreviations:* ATP III = Adult Treatment Panel III; BMI = body mass index; HDL-C = high-density lipoprotein; hsCRP = high-sensitivity C-reactive protein; LDL-C = low-density lipoprotein; MetS = metabolic syndrome.

### The prevalence and components of MetS in Asian geriatrics

According to the 2005 NCEP-ATPIII definition of MetS, the prevalence of MetS was 36.4%. Excluding DM, the prevalence was 27.8%, which was lower than the previously reported data for Caucasians [28]. Because MetS was composed of multiple components and mostly defined from younger aged adult populations, we were interested in which component played the most dominant roles in this geriatric community. The most common diagnostic factor for MetS was hypertension, followed by a large waist circumference, low HDL-C, high fasting glucose, and high triglycerides (Table 1).

### The associations between MetS, inflammation and physical activity

**Figure 1:**
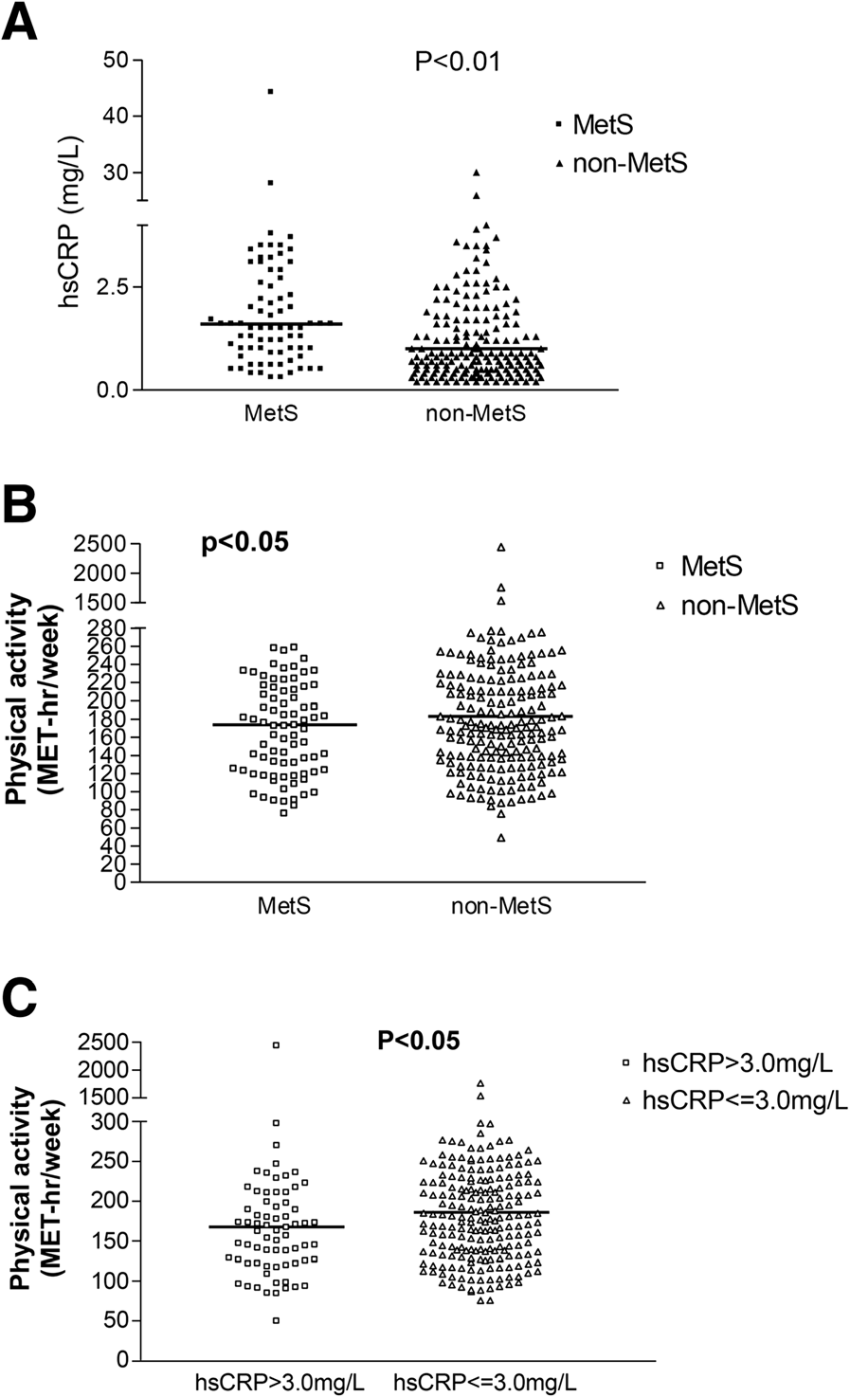
**The associations between MetS, inflammation and physical activity. A**. The level of hsCRP stratified by MetS, hsCRP in MetS: 1.6 (1, 3.2) mg/L, hsCRP in non-MetS: 1.0 (0.5, 2.6) mg/L. **B**. The amount of weekly physical activity stratified by MetS. **C**. The amount of weekly physical activity stratified by the level of hsCRP >3.0 mg/L. hsCRP = high-sensitivity C-reactive protein, MetS = metabolic syndrome.

Among novel inflammatory biomarkers, hsCRP was measured in this Asian geriatric cohort to indicate the individual inflammation status. The median plasma hsCRP level was 1.3 ± 0.3 mg/L in the total population, and was significantly higher in the MetS sub-group than non-MetS sub-group (1.6 ± 0.7 vs. 1.0 ± 0.3 mg/L, *p* < 0.01) (Figure 1A). Furthermore, the risk of elevated hsCRP increased with escalating MetS components (*p* for trend < 0.001). The risk of a diagnosis of MetS was significantly higher for those with the highest when compared with those who having the lowest quartile of hsCRP levels (ORs: 2.767, 95% CI: 1.315-5.821, *p* < 0.01). All these evidence support the pathophysiologic correlation between geriatric MetS and inflammation.

The amount of physical activity recorded from our individual questionnaire was compared and analyzed between different metabolic components as well as the inflammatory biomarkers. The non-MetS group had higher amount of median weekly physical activity (non-MetS vs. MetS group: 183.1 ± 19.0 vs. 173.5 ± 10.6 MET-hr/week, *p* = 0.029) (Figure 1B). The physical activity amount was also higher among those with lower hsCRP levels (higher vs. lower hsCRP: 167.8 ± 38.5 vs. 186.1 ± 14.1 MET-hr/week, *p* = 0.013) (Figure 1C). This implied that physical activity was associated with MetS and the inflammation status indicated by the hsCRP level.

### Individual MetS components and inflammation

**Figure 2:**
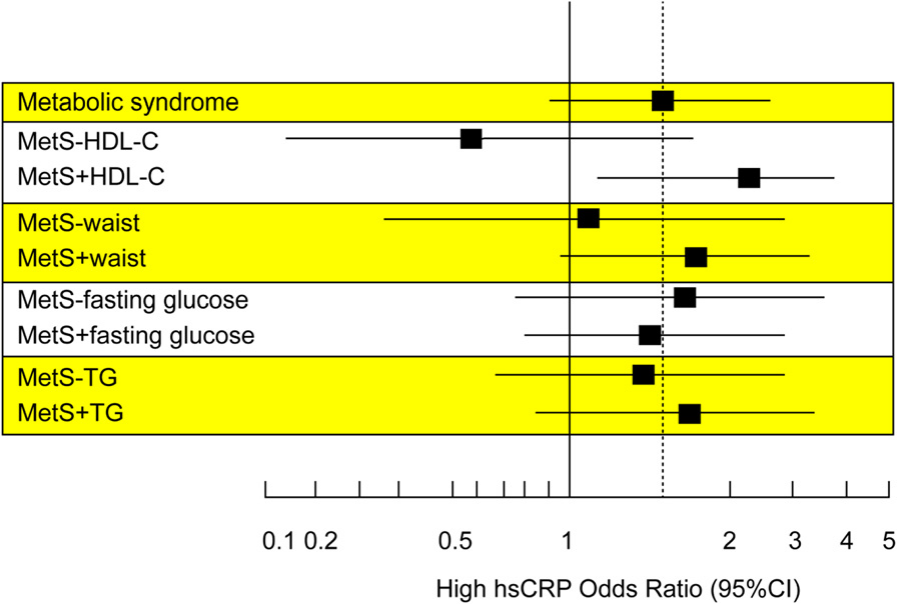
**Interaction between individual MetS components and inflammation.** Odds ratio (95% CI) for high hsCRP plasma levels with MetS, according to absence (−) or presence (+) of single MetS components. HDL-C = high-density lipoprotein, hsCRP = high-sensitivity C-reactive protein, MetS = metabolic syndrome, TG = triglycerides, CI = confidence interval.

To understand the role of plasma hsCRP level in MetS, the participants with MetS were post hoc subdivided into two groups according to their individual MetS components (Figure 2). We used the ORs for the highest hsCRP level from the entire study population with MetS as the standard baseline to analyze the variation between the ORs from those with and without individual MetS risk components. The variation in ORs from MetS participants with-or-without a low HDL-C level was the greatest of the five traditional risk components of MetS. These data indicated that HDL-C level might have the greatest interaction with the plasma hsCRP level. In addition, multivariate analysis with adjustment for physical activity showed that a low HDL-C level was the strongest predictor for higher hsCRP in MetS among all risk components (ORs: 6.318, 95% CI: 1.563-25.536, *p* < 0.05) (Table 2).

**Table 2:** Individual MetS component and physical activity associated with high hsCRP plasma levels in MetS

	**ORs**	**95% CI**	***P***
HDL-C	6.318	1.563-25.536	0.010
Waist	3.031	0.898-10.235	0.074
TG	1.512	0.518-4.412	0.449
Fasting glucose	1.251	0.424-3.692	0.685
Hypertension	1.139	0.215-6.039	0.878
Physical activity	1.000	0.995-1.005	0.967

*Abbreviations:* hsCRP = high-sensitivity C-reactive protein; MetS = metabolic syndrome; ORs = odds ratios; CI = confidence intervals; HDL-C = high-density lipoprotein; TG = triglyceride.

### Individual MetS components and physical activity

**Figure 3:**
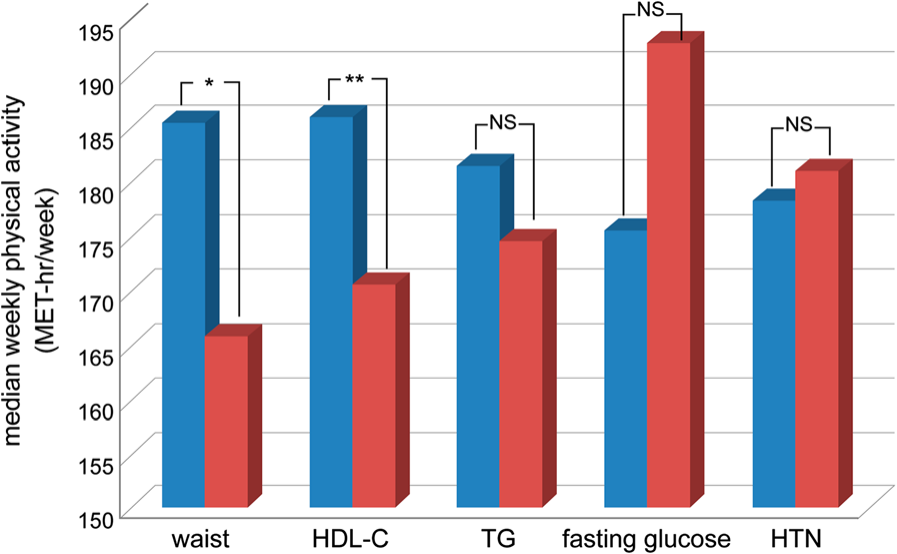
**The median of weekly physical activity stratified by the level of each MetS components.** HDL-C = high-density lipoprotein, HTN = Hypertension, MetS = metabolic syndrome, TG = triglycerides. **: p < 0.01; *: p < 0.05; NS = not significant. (Blue column: positive for individual risk factor; Red column: negative for individual risk factor).

Since the physical activity was associated with the prevalence rate of MetS, we also analyzed its association with individual MetS components (Figure 3). We found that either those with lower waist circumference (higher vs. lower: 165.9 ± 24.8 vs. 185.5 ± 17.2 MET-hr/week, *p* = 0.048) or higher levels of HDL-C (higher vs. lower: 186.1 ± 18.7 vs. 170.6 ± 11.3 MET-hr/week, *p* = 0.003) had a higher median weekly physical activity. But this phenomenon was not significant regarding hypertension, glucose control or triglyceride level.

### Multiple risk factors analysis

Multivariate analysis showed that BMI (ORs: 1.527, 95% CI: 1.319-1.768, *p* < 0.01), insulin (OR: 1.128, 95% CI: 1.045-1.218, *p* < 0.01) and physical activity (ORs: 0.997, 95% IC: 0.994-0.999, *p* < 0.05), rather than the level of hsCRP (ORs: 1.022, 95% CI: 0.965-1.082, *p* > 0.05) were independent predictors of MetS (Table 3).

**Table 3:** Multivariate analysis of physical and biomarker risk factors for MetS among elderly male population

**Factor**	**ORs**	**95% CI**	***P***
BMI	1.527	1.319-1.768	0.001
Insulin	1.128	1.045-1.218	0.002
Physical activity	0.997	0.994-0.999	0.036
LDL-C	0.976	0.946-1.007	0.132
Cholesterol	1.018	0.990-1.046	0.210
Smoking, current	1.639	0.695-3.865	0.259
hsCRP	1.022	0.965-1.082	0.455
Age	1.008	0.954-1.064	0.777

*Abbreviations:* MetS = metabolic syndrome; ORs = odds ratios; CI = confidence intervals; LDL-C = low-density lipoprotein; hsCRP = high-sensitivity C-reactive protein.

## Discussion

Among this elderly community survey, we found an interesting interplay between inflammation and physical activity for the development of MetS. In this geriatric cohort, only the physical activity amount plays the most important role in the pathophysiology of elderly MetS. Though the inflammation may interact with individual risk component in MetS, it remains as a phenomenon associated with the average weekly activity *per se.*

### Association between physical activity and hsCRP level

In this special cohort, we surveyed a remote community with a low level of economic development and low socioeconomic status in southern Taiwan. Most of the residents were farmers whose non-MetS population engaged in substantially more average weekly physical activities than the MetS population. At the same time, they also had lower hsCRP levels, which were generally considered as an indicator of lower inflammatory status.

Previous report showed that physical activity is an effective way to increased human HDL-C [29,30], which was also associated with a higher-level of physical activity as well as hsCRP [11,31]. Indeed, Dai et al. [29] observed another Taiwanese population cohort and found that a high level of physical activity was associated with increased HDL-C levels. Geffken et al. [22] also reported that a high level physical activity might decrease plasma hsCRP level. These data support the notion that physical activity is related to inflammation status, and in our cohort, more physically active participants had lower-grade inflammation biomarkers, i.e., hsCRP levels. Instead of hsCRP, the only remained multivariable predictor for MetS was physical activity amount. We thus hypothesized that inflammation process might be an intermittent phenomenon due to their limited physical activity.

### Lower HDL-C level may interact with hsCRP level in MetS

Our analytic model analyzed the interaction between individual risk components with inflammation among patients with MetS by dividing them into two groups, based on whether or not they had the existing individual MetS component. Interestingly, we found that the variation between risks of higher hsCRP was greatest within the HDL-C level, which indicated that HDL-C level might have the greatest interaction with the plasma hsCRP level in the traditional MetS risk factors. Previous report showed that physical activity is an effective way to increased human HDL-C [29,30], which was also associated with hsCRP [11,31]. But after adjustment for physical activity, the interaction was still remained. ATP-binding membrane cassette transporter A1 (ABCA1) is a possible link between inflammation and reverse cholesterol transport [32]. The function of ABCA1 in modulating the immune response and inflammation works through its direct and indirect antiinflammatory mechanisms including lipid transport, HDL-C formation and apoptosis. These facts support the hypothesis that HDL-C may interact with plasma hsCRP through this ABCA1 pathophysiologic pathway, instead of mechanism from physical activity modulation. Compared with a previous report on a Caucasian cohort [33], this is unique and deserves an additional genetic or a larger cohort survey.

### Central obesity is associated with hsCRP level in MetS

In our study cohort, once we excluded those with a waist circumference ≥ 90 cm, there was no difference in the plasma hsCRP levels between the MetS and non-MetS groups. This analysis implied that waist circumference, or so-called “central obesity”, greatly contributes to the inflammation in MetS. In this geriatric cohort, the variation in ORs from the MetS and non-MetS groups with a waist circumference ≥ 90 cm remained higher (Figure 2), which indicated that central obesity, rather than fasting glucose and triglyceride levels, might also interact with inflammation. The InCHIANTI study [33] showed that waist circumference was the major reason for elevated plasma hsCRP levels. The measurement of waist circumference is commonly recommended as a useful tool for clinically assessing visceral fat accumulation [34,35]. In our study, the median value of the hsCRP level was significantly higher in the MetS group than in the non-MetS group. However, once we excluded those without large waist circumferences, there was no significant difference in the plasma hsCRP level. This subtraction data suggested that central obesity has an important interaction with inflammation, which is indicated by a higher hsCRP level.

### Plasma hsCRP levels vary between Caucasians and Asians

Previous studies [36,37] have suggested hsCRP level may differ by ethnicity. Ye et al. [38] reported one median value of hsCRP level from a Chinese population 50–70 years old and living in Beijing and Shanghai to be 0.68 mg/L, which was lower than the reported [39] Caucasian level. The Study of Women’s Health Across the Nation [37] reported that the median values of hsCRP of Japanese and Chinese women were both also lower than the Caucasian subjects (0.5 mg/L vs. 0.7 mg/L and 1.4 mg/L). Regarding the gender comparison, the report from the Intermountain Heart Collaborative Study Group showed a significant higher mean hsCRP level was observed in American woman (woman vs. man: 1.47 mg/dL vs. 1.30 mg/dL, *p* < 0.001) [40]. In general, the level of hsCRP was higher in Caucasian population and also higher among the women in gender.

Our current study surveyed only gentleman within this remote geriatric community. Compared with prior younger cohort studies, our study showed a relatively higher median level of hsCRP (1.3 mg/L). The higher level of hsCRP could only partially explained by the nature aging process, because aging *per se* has been associated with increased inflammatory status also other degenerative disorders [41]. However, further studies comparing both genders in the same region were mandatory.

### MetS component distribution may vary between different cohorts

The pathophysiological relationship between MetS and its individual components is not well understood. Traditional concepts recognize “central obesity” and “insulin resistance” as the 2 major pathogenic factors [5]. In the InCHIANTI study [33], the most common diagnostic factor was hypertension (28.1%), followed by large waist circumference (22.8%), high triglycerides (17.2%), high fasting glucose (16.3%), and low HDL-C (15.6%). In our study, the most 2 common diagnostic factors were also hypertension (25.9%) and a large waist circumference (21.5%). Interestingly, our third most common MetS diagnostic factor was low HDL-C (20.6%) rather than high triglycerides, which was the least common factor. These data also raise the consideration that various weighting ratios for individual metabolic risk components may be appropriate for different ethnic backgrounds. For example, compared with the InCHIANTI study, a lower level of HDL-C in our study seemed more important than a higher triglyceride level geriatric MetS in the Taiwanese population.

### Limitations

In addition to limited number in our local community study, this unique field study remained limited because of several important confounding and study design factors. First, our study cohort enrolled only men. Whether there are significant gender differences in the pathophysiology of MetS [42] deserves additional investigation. Second, our study participants were all over 65, so the survival effect cannot be excluded. Third, we studied only a rural population. Aekplakorn et al. [43] have shown that MetS affected both urban and rural populations with different patterns of MetS combinations. Fourth, our participants were volunteers but not randomly sampled, which might be biased because of the decreased participation rate of disabled persons. Fifth, we did not screen for their whole dietary habits. Dietary habit may be a main link the difference in prevalence of MetS between Western and Eastern countries. Interestingly, we previously reported another behavior as tea drinking to be a negative predictor for MetS [8]. Culture-associated differences in dietary and daily activity actually play important roles pathophysiologically. Finally, plasma hsCRP was examined only once in our study. It was unknown whether the hsCRP level fluctuated in that elderly population.

## Conclusion

From this remote male cohort data, we demonstrated that Asian geriatric MetS had different metabolic profiles than the Caucasian populations. In addition, we concluded that different from central obesity in Caucasian cohort, the reduced physical activity was the major pathophysiological MetS factor in our Asian geriatric participants.

## Abbreviations

MetS: Metabolic syndrome; haCRP: High-sensitivity C-reactive protein; ORs: Odds ratios; DM: Diabetes mellitus; IPAQ: International physical activity questionnaire; MET-hr/week: Metabolic equivalent task hours per week; NCEP-ATPIII: National cholesterol education program adult treatment panel III; HbA1C: Hemoglobin A1c; HDL-C: High-density lipoprotein; LDL-C: Low-density lipoprotein; BMI: Body mass index; SD: Standard deviation; SE: Standard error; CI: Confidence interval; TG: Triglycerides; HTN: Hypertension; MetSC: Metabolic syndrome component.

## Competing interests

All the authors declared no competing interests.

## Authors’ contributions

CLC, PTL, WTC, CSC and PYL performed the survey and analyzed the data. CLC, CHW, LMT and PYL designed the study survey. CLC and PYL wrote the manuscript. JHC, LMT and PYL reviewed the manuscript. All authors read and approved the final manuscript.
